# Mendelian Susceptibility to Mycobacterial Disease in Egyptian Children

**DOI:** 10.4084/MJHID.2012.033

**Published:** 2012-05-07

**Authors:** Nermeen Galal, Jeannette Boutros, Aisha Marsafy, Xiao-Fei Kong, Jacqueline Feinberg, Jean-Laurent Casanova, Stéphanie Boisson-Dupuis, Jacinta Bustamante

**Affiliations:** 1 Primary Immunodeficiency Clinic, Department of Pediatrics, Cairo University, Egypt; 2 St. Giles Laboratory of Human Genetics of Infectious Diseases, Rockefeller Branch, The Rockefeller University, New York, NY, USA; 3 University Paris Descartes, Necker Medical School, Paris, France; 4 Laboratory of Human Genetics of Infectious Diseases, Necker Branch, Institut National de la Santé et de la Recherché Médicale, U980, Necker Branch, Paris, France

## Abstract

**Background:**

Tuberculosis remains a major health problem in developing countries especially with the emergence of multidrug resistant strains. Mendelian Susceptibility to Mycobacterial Disease (MSMD) is a rare disorder with impaired immunity against mycobacterial pathogens. Reported MSMD etiologies highlight the crucial role of the Interferon gamma /Interleukin 12 (IFN-γ/ IL-12) axis and the phagocyte respiratory burst axis.

**Purpose:**

Screen patients with possible presentations for MSMD.

**Methods:**

Patients with disseminated BCG infection following vaccination, atypical mycobacterial infections or recurrent tuberculosis infections were recruited from the Primary Immune Deficiency Clinic at Cairo University Specialized Pediatric Hospital, Egypt and immune and genetic laboratory investigations were conducted at Human Genetic of Infectious Diseases laboratory in Necker Medical School, France from 2005–2009. IFN-γ level in patient’s plasma as well as mutations in the eight previously identified MSMD-causing genes were explored.

**Results:**

Nine cases from eight (unrelated) kindreds were evaluated in detail. We detected a high level of IFN-γ in plasma in one patient. Through Sanger sequencing, a homozygous mutation in the *IFNGR1* gene at position 485 corresponding to an amino acid change from serine to phenylalanine (S485F), was detected in this patient.

**Conclusion:**

We report the first identified case of MSMD among Egyptian patients, including in particular a new IFNGR1 mutation underlying IFN-γR1 deficiency. The eight remaining patients need to be explored further. These findings have implications regarding the compulsory Bacillus

## Introduction

Tuberculosis (TB) still constitutes a leading cause of mortality and morbidity especially in developing countries. Patients with Mendelian Susceptibility to Mycobacterial Disease (MSMD) present clinical disease caused by weakly pathogenic mycobacteria such as Bacillus Calmette-Guérin (BCG) vaccines and/or nontuberculous environmental mycobacteria in otherwise healthy individuals.^1^ These patients are also vulnerable to *Mycobacterium tuberculosis*.^2^,^3^ MSMD is generally attributed to defects in the Interleukin 12-Interferon gamma (IL-12/IFN-γ) pathways. IFN-γ immunity is important for control of intracellular organisms like *Mycobacterium tuberculosis*, atypical mycobacteria, BCG, and Salmonella. Upon ingestion of these intracellular organisms, the macrophage secretes IL-12, which binds to its receptor (IL12RB1 and IL12RB2) present at the surface of T and NK cells, leading ultimately to IFN-γ secretion by these cells. IFN-γ then binds its receptor (composed of IFNGR1 and IFNGR2) on the macrophage and activates genes including IL-12 and the respiratory burst NADPH oxidase which will allow the macrophage to kill the ingested bacteria.^4^ *IFNGR1* and *IFNGR2*, *STAT1*, *IL12B* and *IL12RB1* are all identified players that are involved in interleukin 12/23-dependent IFN-γ mediated immunity. Human mutations in *IFNGR1*, *IFNGR2* and *STAT1* impair the cellular response to IFN-γ, whereas mutations in *IL12B* and *IL12RB1* impair the IL-12/23-dependent production of IFN-γ.^5^,^6^ More recently, human mutations in *IRF8*, an interferon regulatory factor inducible by IFN-γ, impair IL-12 secretion by monocytes and dendritic cells.^7^ *NEMO* and *CYBB* mutations are responsible of X-linked MSMD.^6^,^8^ We have studied immunologically and genetically patients with disseminated BCG infection following vaccination, atypical mycobacterial infections or recurrent tuberculosis infections collected in Primary Immune Deficiency Clinic at Cairo University Specialized Pediatric Hospital, in Egypt from 2005–2009.

## Patients and Methods

Clinical cases were recruited from Primary Immune Deficiency Clinic at Cairo University Specialized Pediatric Hospital, in Egypt, in the period 2005 from through 2009. Immunological and genetic investigations were conducted at the Human Genetic of Infectious Diseases laboratory in Necker Medical School, France. The study was approved by the institutional review board (IRB) and informed consents were obtained from each patient or the patient’s family. The IRB was also consulted and the study approved in France. Children presenting with the following manifestations were included: BCG infection –culture identification of *Mycobacterium bovis* BCG strain, disseminated BCG infection following vaccination (BCG-osis) inferred on clinical, radiologic findings, atypical mycobacterial infections in immune competent children, mycobacterial disease resistant to therapy (disease that does not respond to two or more standard anti-TB drugs) and microbiological evidence of Salmonella. Patients with acquired or inherited disorders known to cause disseminated BCG infection, such as Severe Combined Immune Deficiency or T cell defects, chronic granulomatous disease, Di George syndrome or AIDS were excluded of this study. All patients were subjected to thorough history taking and systematic clinical examination with emphasis on consanguinity, family history, vaccination history (age, site, route, complications), infection history, system review (e.g. pulmonary, gastrointestinal manifestations), tuberculin test, treatment used, duration, outcome. Immunological assessment included complete blood counts, nitro blue tetrazolium reduction test, T, B and NK cell subsets by flow cytometry (using FACSE Pics, coulter) CD3, CD4, CD8, CD56, CD19, immunoglobulins (Radio immunoassay), and human immunodeficiency virus serology (HIV) status. IFN-γ in plasma was measured by enzyme-linked immunosorbent assay (ELISA) [9]. Genomic DNA was extracted from whole blood as described previously.^10^ The coding and flanking sequence of 8 genes known to predispose to MSMD were amplified and sequenced with an ABI 3130x sequencer. The pairs of primers and PCR conditions are available upon request. Polymorphism phenotyping (PolyPhen) was used to predict the possible impact of an amino acid substitution on the structure and the function of a human protein.

## Results

Fifteen children with disseminated mycobacterial infection were initially included. Six of them were excluded after their immunological workup analysis identified Primary Immunodeficiency disorders including Severe Combined Immunodeficiency (n=5) and Chronic Granulomatous Disease (n=1). We investigated the eligible case series which included five males and four females. The mean patient age was 3.5 years ± 4.9. The mean age of presentation was 3.2 months. Consanguinity was positive in six out of nine cases (first degree). Two families had history of previous sibling deaths (due to similar conditions) and one mother had history of two previous abortions (**Table 1**). All patients received BCG vaccine during the first two months of life (except number 9 whose vaccination was withheld because of sibling death (due to mycobacterial diseases). The vaccine was administered intradermally, in the upper left arm by registered health workers according to the national vaccination policy. Seven out of the nine cases reported local complications related to BCG in the form of poor healing, ulceration, abscess formation, skin tuberculosis (lupus vulgaris-like) and/or local lymphadenitis. Clinical presentations included for case 1, lymphadenopathy caused by an atypical mycobacterial (*Mycobacterium fortuitum*) (**Table 1**), for case 2, recurrent bloody diarrhea caused by *Salmonella enteritidis* infection, case 3 had mycobacterial osteomyelitis fistulizing to bone, and case 8 had renal tuberculous granulomata evidenced by renal biopsy. Presentations and demographic data are shown in **Table 1**. Generalized lymphadenopathy was present in five cases (n=5) whereas the chest was involved in four cases (n=4). All patients received antituberculous medications in the form of triple therapy including Isoniazide, Rifampicin, Pyrazinamide (with exchange of Pyrazinamide with Streptomycin in cases of BCGosis) and or quadruple therapy (cases number 3 and 8) (Ethambutol/ Dalacin) with good response in five cases. Treatment was set initially for six months and extended according to the progress of cases individually. As for outcome, three patients (cases # 3, 4 and 6) died of complications (fulminant Pneumoniae), one of patients remains symptomatic with lymphadenopathy that is progressing despite therapy (case # 2) and five patients cleared their infections after prolonged therapy .

We measured IFN-γ in plasma of nine patients and we found a high IFN-γ plasma levels (813 pg/ml) in one patient (patient # 3) highly suggestive of a complete IFNGR deficiency. The 7 coding and flanking intron sequences of *IFNGR1* were amplified and sequenced. We identified a homozygous mutation in exon 7 of *IFNGR1* gene leading to the replacement of a serine at amino acid position 485 to phenylalanine (S485F), probably responsible for complete IFNGR1 deficiency and the MSMD syndrome. Unfortunately, this patient died and we do not dispose the cells to characterize the impact of this variation. Indeed, the variation has not previously been reported in any SNP database, the change is non-conservative and affects an amino-acid relatively conserved among species. We carried out bioinformatics analysis by PolyPhen; this method predicted that S485F is “probably damaging” with a score of 0.99 of protein structure. Moreover the C-terminal part of IFNGR1 had been proved to be essential for the docking of STAT1 upon activation. No other mutations have been identified in the seven other genes of MSMD in this patient. Indeed, the combination of high IFN-γ in plasma and the mutation’s impact predicted are sufficient for the diagnosis. Eight other patients included in this work presented wild type sequences in the exons of eight genes involved in MSMD.

## Discussion

This study reports the first identified case of MSMD among Egyptian children. Other colleagues have previously studied the association of interferon-gamma (IFN-γ) and interleukin-10 (IL-10) single nucleotide polymorphisms with tuberculous infection and post-BCG lymphadenitis in Egyptian children revealing that low producer IFN-γ +874 A/A genotype was associated with post-BCG lymphadenitis and TB disease especially in younger children below 5 years.^11^ Older studies have looked into the relation between atypical mycobacterial infections and mammalian tuberculosis perhaps describing an unrecognized MSMD back then.^12^

In our series, the case with IFN-γR1 deficiency was BCG vaccinated at seven weeks of age, manifested at four months and suffered extensive spreading lesions over the course of several years highlighting the prolonged course patients may suffer. She was diagnosed and treated as a case of extensive tuberculosis. The early age of presentation necessitates increasing physicians’ awareness to initiate early treatment and requires vigilance for more aggressive therapy if required.

Consanguinity was positive in six out of nine cases in the series, high rates of consanguinity and in –breeding may lead to occurrence of recessive disorders at higher rates than other populations. The average consanguinity rate in Egypt which was estimated to be 28.96% in the eighties has risen recently to thirty-forty percent with an average inbreeding coefficient of 0.010.^13^,^14^ Interestingly, the first patient with MSMD was reported in Tunisia, a country with a similar pattern of inbreeding and consanguinity.^15^ Since MSMD disorders may be transmitted either in a recessive or dominant manner, there is a growing potential for the existence of recessive forms. A study of inbred consanguineous populations from North African countries recommended investigating IL-12RB1 deficiency in patients with severe tuberculosis whether or not adverse effects of BCG vaccination or atypical mycobacteriosis were observed.^16^ One of the cases suffered recurrent attacks of Salmonella enteritidis, a finding reported in several other studies.^17^,^18^

The remaining eight cases might have had unrecognized defects of NADPH axis as it is also important for the control of mycobacterial infections. More genetic mutations of IFN-γ pathways are discovered every day helping to elaborate their role in disease causation and the magnitude of the problem. MSMD exists in Egyptian children and needs to be explored further. The remaining cases will undergo combination of exome-sequencing and linkage analysis to elucidate the genetic disease.^19^,^20^

## Conclusion

MSMD exists in Egyptian children; finding should have implications on the compulsory BCG vaccination policy especially with the high consanguinity rates, deferring vaccination till further evaluation occurs.

## Figures and Tables

**Table 1.** Characteristics of the study group

**Case number**	**1**	**2**	**3**	**4**	**5**	**6**	**7**	**8**	**9**
**Age** (Year)	0.5	4	16	1.5	2	1.5	1	5	0.3
**Sex (M/F)**	F	M	F	M	M	F	M	F	M
**Consanguinity**	−	**+**	**+**	**+**	−	+	−	+	+
**Similar conditions**	−	2 sib deaths	2 maternal abortions	−	−	2 sib deaths	−	−	3 sib deaths
**Presentation**	BCG osis	BCGosis Diarrhoe GLN	BCGosis Fistulizing bone lesions GLN Lung	BCGosis Diarrhoea FTT GLN Lung	Atypical mycobacteri a GLN	BCGosis GLN Lung FTT	BCGosis	BCGosis GLN Renal granulom− as	Lung
**Age of onset (month)**	5	4	2	4	4	2	3	3	4
**Mycobacterial strain**	BCG	BCG	BCG	BCG	NTM	BCG	BCG	BCG	TB
**Viral infections**	−		−	−	−	−	−	−	−
**Salmonella**	−	**+**	−	−	−	−	−		−
**Vaccination**	**+**	**+**	**+**	**+**	**+**	**+**	**+**	**+**	−
**Response to therapy**	**G**	**R**	**R**	**R**	**G**	**R**	**G**	**G**	**G**
**Outcome**	**AW**	**AS**	**D**	**D**	**AW**	**D**	**AW**	**AW**	**AW**
**Identified mutations**	−	−	**IFNGR1**	−	−	−	−	−	−

M=male, F=female, + =positive, – =negative, BCGosis (disseminated BCG infection following vaccination), GLN=Generalized lymphadenopathy, FTT=Failure to thrive, TB=tuberculous, NTM=Nontuberculous mycobacteria G=Good, R=Resistant, AW=Alive and well, AS=Alive and symptomatic, D=Deceased, IFNGR1 =Interferon gamma receptor 1 deficiency
